# Health Disparities in Hepatitis C Screening and Linkage to Care at an Integrated Health System in Southeast Michigan

**DOI:** 10.1371/journal.pone.0161241

**Published:** 2016-08-15

**Authors:** Kassem Bourgi, Indira Brar, Kimberly Baker-Genaw

**Affiliations:** 1 Department of Internal Medicine, Henry Ford Hospital, Detroit, MI, United States of America; 2 Division of Infectious Diseases, Henry Ford Hospital, Detroit, MI, United States of America; Public Health Agency of Canada, CANADA

## Abstract

With recommended screening for hepatitis C among the 1945–1965 birth cohort and advent of novel highly effective therapies, little is known about health disparities in the Hepatitis C care cascade. Our objective was to evaluate hepatitis C screening rates and linkage to care, among patients who test positive, at our large integrated health system. We used electronic medical records to retrospectively identify patients, in the birth cohort, who were seen in 21 Internal Medicine clinics from July 2014 to June 2015. Patients previously screened for hepatitis C and those with established disease were excluded. We studied patients’ sociodemographic and medical conditions along with provider-specific factors associated with likelihood of screening. Patients who tested positive for HCV antibody were reviewed to assess appropriate linkage to care and treatment. Of 40,561 patients who met inclusion criteria, 21.3% (8657) were screened, 1.3% (109) tested positive, and 30% (30/100) completed treatment. Multivariate logistic regression showed that African American race, male gender, electronic health engagement, residency teaching clinic visit, and having more than one clinic visit were associated with higher odds of screening. Patients had a significant decrease in the likelihood of screening with sequential interval increase in their Charlson comorbidity index. When evaluating hepatitis C treatment in patients who screened positive, electronic health engagement was associated with higher odds of treatment whereas Medicaid insurance was associated with significantly lower odds. This study shows that hepatitis C screening rates and linkage to care continue to be suboptimal with a significant impact of multiple sociodemographic and insurance factors. Electronic health engagement emerges as a tool in linking patients to the hepatitis C care cascade.

## Introduction

Hepatitis C affects 185 million people worldwide[1] and over 3.2 million Americans[2, 3]. Hepatitis C is associated with high morbidity, frequent complications, and a higher mortality rate than HIV [4]. Direct-acting antivirals (DAA) can cure over 95% of hepatitis C infections through short-course, well-tolerated regimens [5]. With DAA availability, hepatitis C eradication becomes dependent on optimizing screening and linkage to care [6]. Experience in treating HIV patients has refined understanding of “linkage to care” and “care cascade” concepts whereby patients are screened, treated, and followed for adherence and response [7]. These concepts are also applicable in hepatitis C care [8].

As the 1945–1965 birth cohort has the highest hepatitis C prevalence [9], the Centers for Disease Control and Prevention and US Preventive Services Task Force recommended they receive 1-time hepatitis C testing regardless of risk factors [10, 11]. In previous studies screening has been as low as 1%-16% in eligible patients [12] and less than half of those positive underwent hepatitis C RNA testing [13–16], the first step in the hepatitis C care cascade. For our large health system in southeast Michigan, we aimed to identify hepatitis C screening rates and linkages to care in patients who test positive for hepatitis C antibody.

## Materials and Methods

This retrospective cohort study identified patients born between 1945 and 1965 who were seen at any internal medicine (IM) clinic within the Henry Ford Health System from July 1, 2014 through June 30, 2015. Based in Detroit, Henry Ford Health System serves a population of over 4 million in southeast Michigan. The study was conducted at 21 urban and suburban general internal medicine practice clinics serving an insured population of approximately 100,000 patients. One-fourth of these clinics are part of the IM residency teaching program while the rest are staffed by Henry Ford Medical Group physicians. All enrolled clinics included hepatitis C antibody screening as one of the health maintenance modules in the EMR. The module reminded age-appropriate patients, through emails, and their providers, through programmed alerts at the time of the office visit, that they are due for screening. This was implemented 3 months prior to our study’s start date and was a part of the comprehensive health maintenance section in the EMR. No separate educational interventions, pertinent to hepatitis C screening, were conducted prior to or during the study period.

This study aimed to identify rates of hepatitis C screening, linkage to care, and completed treatment in patients of the 1945–1965 birth cohort. The study was approved by the Henry Ford Health System Institutional Review Board and the requirement for an informed consent was waived. All patient records and pertinent information were anonymized and de-identified prior to the analysis.

Using the electronic medical record (EMR) data repository, we identified all patients in the birth cohort who had at least 1 IM clinic visit during the study period. Patients with previous diagnosis of chronic hepatitis C were excluded, as were patients who had already been screened for hepatitis C prior to their clinic visit. We identified patients’ sociodemographic factors, clinical comorbidities, engagement in the EMR patient portal, as well as provider-specific factors to assess their association with the likelihood of hepatitis C screening. We then reviewed the charts of all patients who screened positive for hepatitis C antibody to assess for appropriate linkage to care and treatment. The care linkage dataset contained demographic information, dates and locations of all clinic visits, and results of laboratory studies. In assessing hepatitis C treatment rates, we excluded patients with active drug or alcohol use as they were ineligible for treatment due to insurance restrictions for active users.

Demographics included date of birth, gender, race, income level, and geographic site of clinic visit. Income level was estimated based on the average household income for the patient location according to geocode census from public census data. The history of drug use was extracted from the medical records and was contingent on patients’ self-reporting as well as physicians’ documentation. Electronic health engagement was defined as active subscription, through accessing the patient portal of the EMR within the last 1 year. To assess the degree of liver fibrosis among patients diagnosed with hepatitis C, we used the noninvasive FIB-4 score which combines patient’s age with biochemical values (liver enzymes and platelet counts). A FIB-4 score of <1.45 has a negative predictive value of 94.7% for severe fibrosis while a score of >3.25 has a positive predictive value of 82.1% for severe fibrosis [17].

### Statistical analysis

We used descriptive statistics, chi-square analysis, two-sample t-tests, and Wilcoxon rank sum tests to identify the proportion of patients screened for hepatitis C, patient and provider factors associated with screening, hepatitis C positivity rate, and factors associated with appropriate linkage to care among hepatitis C antibody positive patients. Categorical variables were compared using chi-squared test of independence and numeric variables were compared with two-sample t-test or Wilcoxon rank sum. We also performed a multivariate logistic regression to analyze the odds of ever being screened and then being treated if they tested positive. Variables significant in univariate analysis and confounders with a p-value < 0.25 were included in the multivariable model, thereby helping to ensure adequate power and stability. On multivariate analysis, we divided the Charlson comorbidity index into 3 categories: index scores of ≥ 4, 2–3, and ≤ 1. All statistical tests were conducted at a two-sided significance level of 0.05.

## Results

A total of 47,304 patients in the birth cohort were seen in our IM clinics during the 1-year study period. After excluding 6743 patients who were previously screened or had established hepatitis C disease, 40,561 patients met inclusion criteria. Of these, 8657 patients (21.3%) were screened for hepatitis C, using HCV antibody test, and 109 (1.3%) tested positive.

### Patient characteristics

Patient demographics are shown in Table 1. Patients had a mean age of 59.4 ± 5.8 years and were primarily female (59.1%), Caucasian (50.5%) and African American (43.0%). The patients had a mean of 2.2 clinic visits (S1 Table) within the study period with 17.6% of the patients seen in the IM residency teaching clinics. Current or previous history of drug use was reported in 1415 patients (3.5%). Distribution of the median of median household income for the birth cohort is shown in S2 Table.

**Table 1:** Birth Cohort Demographic Characteristics.

Variable	Result
**Age, years** (± SD)	59.4 ± 5.8
**Race**	
African American	15,154 (43.0%)
Caucasian	17,783 (50.5%)
Other	2306 (6.6%)
**Gender**	
Male	16,585 (40.9%)
Female	23,976 (59.1%)
**HIV positive**	164 (0.4%)
**Drug use (current or former)**	1415 (3.5%)
**Clinic setting**	
Residency teaching clinics	7148 (17.6%)
Other practice	33,413 (82.4%)
**Average number of office visits in 12 months** (± SD)	2.2 ± 1.6
**Median household income** (± SD)	$52,037 ± 20,365
**Average Charlson Comorbidity Index** (± SD)	0.88 ± 1.5

SD = standard deviation

### Screening for hepatitis C

Univariate analysis results are shown in Table 2. African Americans were more likely to be screened than Caucasians (23.3% vs. 19.8%, *P* < 0.001). Men were more likely to be screened than women (22.2% vs. 20.7%, *P* < 0.001). Patients engaged in electronic health also had higher screening rates (22.7% vs. 19.8%, *P* < 0.001). Screening was more common in patients seen in residency teaching clinics (23.5% vs. 20.9%, *P* < 0.001), and screened patients had a higher mean number of visits (2.36 vs. 2.14, *P* < 0.001). Patients who were screened for hepatitis C had a slightly lower, but not statistically significant, mean Charlson comorbidity index (0.86 vs. 0.91, *P* = 0.88).

**Table 2:** Univariate analysis comparing screened versus not screened.

Variable	Screened N = 8657	Not Screened N = 31,904	P-Value
**Age, years** (± SD)	59.5 ± 5.7	59.3 ± 5.9	*0*.*007*
**Gender**			*<0*.*001*
Male	3684 (22.2%)	12,901 (77.8%)	
Female	4973 (20.7%)	19,003 (79.3%)	
**Race**			*<0*.*001*
Caucasian	3527 (19.8%)	14,256 (80.2%)	
African American	3538 (23.3%)	11,616 (76.7%)	
Other	1592 (20.9%)	6032 (79.1%)	
**Electronic health engagement**			*<0*.*001*
Subscribed to patient portal	4929 (22.7%)	16,769 (77.3%)	
Non-subscribers	3728 (19.8%)	15,135 (80.2%)	
**Clinic setting**			*<0*.*001*
Residency teaching clinic	1677 (23.5%)	5471 (76.5%)	
Other clinics	6980 (20.9%)	26,433 (79.1%)	
**Drug use (current or former)**	1104 (78%)	311 (22%)	*0*.*552*
**Average number of office visits in 12 months** (± SD)	2.36 ± 1.66	2.14 ± 1.55	*<0*.*001*
**Median household income** (± SD)	$52,146 ± 20,766	$52,008 ± 20,256	*0*.*89*
**Average Charlson Comorbidity Index** (± SD)	0.86 ± 1.38	0.91 ± 1.51	*0*.*88*

SD = standard deviation

Multivariate logistic regression results are shown in Table 3 and all variables included in the multivariate model are in S3 Table. These analyses reinforced that African American race (adjusted OR [aOR] 1.34, 95% CI 1.25–1.34) and male gender (aOR 1.18, 95% CI 1.11–1.25) were associated with higher odds of screening. Similarly, odds of screening were higher in patients engaged in electronic health (aOR 1.24, 95% CI 1.17–1.31), those seen within a residency teaching clinic (aOR 1.20, 95% CI 1.11–1.30), and patients seen more than once in IM clinics during the study period (aOR 1.42, 95% CI 1.34–1.51). Patients had a significant decrease in the likelihood of screening with each sequential interval increase in their Charlson comorbidity index (aOR 0.87, 95% CI 0.82–0.92).

**Table 3:** Variables associated with hepatitis C virus screening on multivariate analysis.

Variable	Odds Ratio	95% CI	P-Value
**African American race**	1.34	1.25–1.43	*<0*.*001*
**Male gender**	1.18	1.11–1.25	*<0*.*001*
**Electronic health engagement**	1.24	1.17–1.31	*<0*.*001*
**Office visits**			
Setting: residency teaching clinic	1.20	1.11–1.30	*<0*.*001*
Frequency > 1 office visit	1.42	1.34–1.51	*<0*.*001*
**Interval Charlson Comorbidity Index**	0.87	0.82–0.92	*<0*.*001*
**Drug use (current or former)**	1.01	0.88–1.17	*0*.*87*

### Linkage to care

Of the 109 patients who tested positive for hepatitis C antibody, 5 (4.6%) had active drug or alcohol use and were excluded from treatment analysis. Of the remaining 104 patients, 69 patients (66.4%) had quantitative hepatitis C RNA testing and 51 patients (49%) were evaluated by a hepatitis C specialist (Infectious Diseases or Gastroenterologists). Baseline demographic and clinical characteristics of patients who screened positive for hepatitis C antibody and RNA test are shown in S4 Table. A total of 30 patients completed the course of hepatitis C treatment, 70 patients were not treated, and 4 patients did not require any therapy as they had undetectable hepatitis C virus RNA. Table 4 summarizes the proportions of treated and untreated patients categorized by patient characteristics. Likelihood of treatment was significantly higher in women (42.1% vs. 22.6%, *P* < 0.05) but did not differ by race. There was a trend towards a lower treatment rate in patients with income levels lower than the state’s mean household income (27% vs. 54.5%, *P* = 0.06). Medicaid beneficiaries were significantly less likely to be treated than Medicare and commercial insurances (10% vs. 35%, *P* < 0.05). Similar to the screening rates, patients engaged in electronic health had a higher likelihood of treatment (45.2% vs. 23.2%, *P* < 0.05).

**Table 4:** Univariate analysis comparing hepatitis C virus positive patients who received treatment to untreated subjects.

Variable	Treated Patients N = 30	Untreated Patients N = 70	P-Value
**Age, years** (± SD)	62.2 ± 4.7	61.1 ± 4.7	*0*.*28*
**Gender**			*0*.*04*
Male	14 (22.6%)	48 (77.4%)	
Female	16 (42.1%)	22 (57.9%)	
**Race**			*0*.*38*
African American	21 (28.0%)	54 (72.0%)	
Other	9 (36.0%)	16 (64.0%)	
**Insurance**			*0*.*03*
Medicaid	2 (10.0%)	18 (90.0%)	
Other Insurance Coverage	28 (35.0%)	52 (65.0%)	
**Electronic health engagement**			*0*.*03*
Subscribed to patient portal EMR	14 (45.2%)	17 (54.8%)	
Non-subscribers	16 (23.2%)	53 (76.8%)	
**Income**			*0*.*06*
Lower than state mean household income	24 (27.0%)	65 (73.0%)	
Higher than state mean household income	6 (54.5%)	5 (45.5%)	
**Clinic setting**			*0*.*93*
Residency teaching clinic	16 (29.6%)	38 (70.4%)	
Other clinics	14 (30.4%)	32 (69.9%)	
**Medical state**			
Mean Fibrosis score (FIB-4)	2.48 ± 2.15	2.37 ± 1.78	*0*.*94*
Severe Fibrosis (FIB-4 > 3.25)	6 (20.7%)	9 (14.5%)	*0*.*46*
Charlson Comorbidity Index	1.77 ± 1.01	1.46 ± 1.71	*0*.*04*

EMR = electronic medical records; SD = standard deviation

Results of multivariate logistic regression are shown in Table 5 and all variables included in the multivariate model in S5 Table. The multivariate model included all variables from Table 4 with a p-value < 0.25. Electronic health engagement was associated with higher odds of treatments (aOR 3.89, 95% CI 1.31–11.54) while Medicaid insurance coverage was associated with significantly lower treatment odds (aOR 0.16, 95% CI 0.16–0.97) compared to patients with different insurance coverage.

**Table 5:** Factors associated with treatment in hepatitis C virus positive patients.

Variable	Odds Ratio	95% CI	P-Value
Female gender	2.36	0.90–6.25	*0*.*08*
Electronic health engagement	3.89	1.31–11.54	*0*.*01*
Medicaid insurance	0.16	0.16–0.97	*< 0*.*05*
Charlson Comorbidity Index	1.10	0.78–1.56	*0*.*58*

## Discussion

The hepatitis C screening rate of 21.3% and completed treatment rate of 30% for the 1945–1965 birth cohort in the IM clinic setting continue to be suboptimal despite the current recommendations. In our study Medicaid beneficiaries were significantly less likely to be treated. Men had higher screening rates but were significantly less likely to be treated. African Americans were more likely to be screened than Caucasians but no racial differences occurred in treatment. Patients engaged in electronic health were the sole group significantly more likely to be screened and treated for hepatitis C.

Our study’s screening rate was higher than rates of 4.3% [18], 12.7% [19], and 15.8% [12] reported by studies conducted before the universal hepatitis C screening recommendations for the birth cohort. The rate of hepatitis C virus RNA testing among patients who screened positive was comparable to reported results from other studies [13–15]. Other studies done in the birth cohort after the Centers for Disease Control and Prevention recommendations also reported higher hepatitis C screening rates. A New York primary care study reported improvement in hepatitis C screening from 11% to 46% after instituting automatic hepatitis C testing, but they did not report on those treated [20]. A safety-net hospital in Texas instituted an EMR-embedded hepatitis C testing algorithm with opt-out consent plus educational interventions for staff, leading to a 49% inpatient screening rate, but only 4% (5/129) started treatment largely because of the high number of uninsured [21].

We analyzed our cohort to identify disparities in hepatitis C screening and treatment. Of our 30 patients who completed hepatitis C treatment, only 2 had Medicaid. While the type of insurance coverage did not influence the likelihood of screening, Medicaid beneficiaries were significantly less likely to receive hepatitis C treatment, if they tested positive. Similar findings were reported in a prospective cohort study assessing the determinants of denial of DAA prescriptions in 4 Northeastern states as prescriptions were more commonly denied for patients with Medicaid than Medicare (46% vs. 4.9%, *P* < 0.001, aOR = 8.97). The median time to DAA prescription fill was also longer for Medicaid patients compared to Medicare or commercial insurance (23 days vs. 14 days, *P* < 0.001) [22]. In a study conducted in Illinois, only 50% of Medicaid patients were approved for DAA therapy, with a median provider time of 92.5 minutes spent to obtain prior authorization [23]. A significant interstate heterogeneity was observed in a systematic evaluation of state Medicaid reimbursement of hepatitis C therapies (sofosbovir) in the United States [24]. Approximately 88% of the state Medicaid committees included absence of active drug or alcohol use in their eligibility criteria and most states restricted hepatitis C drug coverage to patients with advanced fibrosis or cirrhosis [24, 25].

Our patients’ income level, based on geocode estimations, had no effect on hepatitis C screening rates. However, analysis of hepatitis C patients who received treatment showed a trend toward a lower likelihood of treatment in patients with lower income levels. Noticeably, 89% of hepatitis C positive patients had an average household income lower than $50,000, the estimated mean household income in the state of Michigan. This is congruent with previous reports showing that a disproportionate number of persons living with hepatitis C in the United States have a low income [24, 26].

Of the insured, those most likely to receive hepatitis C screening and treatment in our study were engaged in electronic health. Our EMR includes a patient portal that links patients at multiple steps in their care cascade, from reminding patients, through emails, that they are due for health maintenance tests, to allowing patients access to their results, to electronically scheduling follow-up appointments and to communicating with their health care providers, nurses and physicians. Our results are consistent with previous studies showing that the patient portals improve clinical outcomes and disease control in multiple chronic conditions [27–29]. The portal enhances communication between the patients and their health care providers, allowing patients to better understand and manage their disease [30].

Despite the promising role of patient portals, research has demonstrated that patients’ electronic health engagement is largely dependent on personal factors such as age, race, income, and educational levels [31]. This may result in exclusion of vulnerable populations with significantly lower access. Disparities in the use of the patient portal may potentially reinforce and amplify existing health disparities; further studies are needed to evaluate potential barriers to electronic engagement in the underserved patient population.

As the group with the lowest income in the United States [32], African Americans also have the highest hepatitis C prevalence at 22% [33, 34]. In our cohort African Americans were more likely to be screened for hepatitis C. Once diagnosed, there was no difference in likelihood of treatment between African Americans and Caucasians (28% vs. 3%, *P* = 0.38). Hepatitis C risk factors and disease burden vary by ethnicity [35]. A retrospective analysis of a multicenter clinical trial conducted at 118 medical centers in the United States showed that African Americans were 65% less likely to be treated for hepatitis C compared to other races [36]. They were also less likely to respond to traditional hepatitis C therapy (peginterferon plus ribavirin) [33, 37–39]. The higher screening rates seen in African Americans likely reflect providers’ perception of higher disease prevalence in this population. Similar findings were seen in a study evaluating hepatitis C screening at 4 primary care sites in Philadelphia where African Americans without reported hepatitis C risk factors were 54% more likely than Caucasians to be screened for hepatitis C [34].

Men were another group significantly more likely to be screened in our study; however, they were less likely to be treated. When adjusting for confounders, men still had a significantly higher screening rate, while likelihood of treatment was not different. Men may be screened more often as they are likely perceived to be at a higher risk for hepatitis C acquisition. In the National Health and Nutrition Examination Survey from 2003 to 2010, men were 1.7 times more likely than women to have chronic hepatitis C infection [40].

Our study has limitations. As a retrospective chart review, the variables studied were dependent on patient reporting and proper documentation. The data assessing use and sexual history were based upon patient reporting and provider input. Additionally, income level was based on geocoding and not on individual patient reporting. Furthermore, with the retrospective analysis we were unable to address causality between the different variables assessed and screening or treatment rates. Consequently, an in-depth assessment of the observed disparities in both screening and treatment of hepatitis C is limited and will require further studies to be better explicated. Although multiple IM clinic locations with a diverse patient population were included in this study, all data represent a single heath care system. Furthermore, our clinic patients are all insured and therefore we are unable to account for the likelihood of treatment and screening among uninsured subjects.

Our study highlights multiple challenges encountered in the hepatitis C care cascade. Only a small percentage of patients eligible for testing were screened for hepatitis C with a significant influence of sociodemographic and provider-specific factors. Furthermore, patients who tested positive had inadequate linkage to care, particularly Medicaid beneficiaries. Despite the recent progress and success of hepatitis C eradication with the novel DAA therapies, successful control of hepatitis C requires further public health interventions to increase screening rates and access to care. Our study accentuates a promising role for patient engagement in electronic health portals as a tool in linking patients to the hepatitis C care cascade. Hence, continued efforts are needed to increase and improve patient electronic health engagement.

## Supporting Information

S1 Table Number of Office Visits in the Study Period. (DOCX)

S2 Table Distribution of Median Household Income for the Birth Cohort. (DOCX)

S3 Table Full Multivariate Analysis on Variables Associated with Hepatitis C Virus Screening. (DOCX)

S4 Table Characteristics of Patients who Screened Positive for Hepatitis C Antibody Test. (DOCX)

S5 Table Full Multivariate Analysis on Variables Associated with Hepatitis C Linkage to Care. (DOCX)
