# Supplementary material for: Health Disparities in Hepatitis C Screening and Linkage to Care at an Integrated Health System in Southeast Michigan
Source: PLoS One. 2016 Aug 15;11(8):e0161241. doi: 10.1371/journal.pone.0161241 (PMC4985134; doi:10.1371/journal.pone.0161241)
Supplement: S1 Table — (DOCX) [file pone.0161241.s001.docx]

**S1 Table. Number of Office Visits in the Study Period**

| **Number of Office Visits** | **Result** |
| --- | --- |
| 1 visit | 17,351 (44.3%) |
| 2 visits | 10,065 (25.7%) |
| 3 visits | 5,614 (14.3%) |
| 4 visits | 2,893 (7.4%) |
| More than 4 visits | 3,254 (8.3%) |
