# Supplementary material for: Health Disparities in Hepatitis C Screening and Linkage to Care at an Integrated Health System in Southeast Michigan
Source: PLoS One. 2016 Aug 15;11(8):e0161241. doi: 10.1371/journal.pone.0161241 (PMC4985134; doi:10.1371/journal.pone.0161241)
Supplement: S2 Table — (DOCX) [file pone.0161241.s002.docx]

**S2 Table. Distribution of Median Household Income for the Birth Cohort**

| **Median Household Income** | **Result** |
| --- | --- |
| Less than $24,999 | 2,773 (7.1%) |
| $25,000 − $49,999 | 18,527 (47.3%) |
| $50,000 − $74,999 | 12,466 (31.8%) |
| $75,000 − $100,000 | 4,343 (11.1%) |
| More than $100,000 | 1,068 (2.7%) |
