# Supplementary material for: Health Disparities in Hepatitis C Screening and Linkage to Care at an Integrated Health System in Southeast Michigan
Source: PLoS One. 2016 Aug 15;11(8):e0161241. doi: 10.1371/journal.pone.0161241 (PMC4985134; doi:10.1371/journal.pone.0161241)
Supplement: S3 Table — (DOCX) [file pone.0161241.s003.docx]

**S3 Table. Full multivariate analysis on variables associated with hepatitis C virus screening**

| **Variable** | **P-value** | **Odds Ratio** | **Odds Ratio 95% Confidence Limits** | |
| --- | --- | --- | --- | --- |
| **Male Gender** | *<0.001 ** | 1.18 | 1.11 | 1.25 |
| **Electronic Health Engagement** | *<0.001 ** | 1.24 | 1.17 | 1.31 |
| **HIV** | *0.07* | 0.61 | 0.35 | 1.04 |
| **Drug Use** | *0.87* | 1.01 | 0.88 | 1.17 |
| **Residency Teaching Clinic** | *<0.001 ** | 1.20 | 1.11 | 1.30 |
| **More than 1 Office visit** | *<0.001 ** | 1.42 | 1.34 | 1.51 |
| **Interval Charlson Comorbidity Index** | *<0.001 ** | 0.87 | 0.82 | 0.92 |
| **Median Income (in Thousands)** | *<0.001 ** | 1.00 | 1.00 | 1.01 |

* Statistically Significant, P < 0.05
