# Supplementary material for: Health Disparities in Hepatitis C Screening and Linkage to Care at an Integrated Health System in Southeast Michigan
Source: PLoS One. 2016 Aug 15;11(8):e0161241. doi: 10.1371/journal.pone.0161241 (PMC4985134; doi:10.1371/journal.pone.0161241)
Supplement: S4 Table — (DOCX) [file pone.0161241.s004.docx]

**S4 Table. Characteristics of Patients who Screened Positive for Hepatitis C Antibody Test**

| **Variable** | **Result** |
| --- | --- |
| **Age, years** (± SD) | 61.4 ± 4.7 |
| **Age Distribution** |  |
| 50 – 54 | 10 (10%) |
| 55 – 59 | 21 (21%) |
| 60 – 64 | 41 (41%) |
| 65 – 69 | 28 (28%) |
| **Race** |  |
| African American | 75 (75.8%) |
| Caucasian | 15 (15.2%) |
| Other | 9 (9.1%) |
| **Gender** |  |
| Male | 62 (62%) |
| Female | 38 (38%) |
| **Median Household Income** |  |
| Less than $24,999 | 27 (27%) |
| $25,000 − $49,999 | 62 (62%) |
| $50,000 − $74,999 | 8 (8%) |
| More than $75,000 | 3 (3%) |
| **Fibrosis Score (FIB 4)** |  |
| Less than 1.45 | 30 (33.0%) |
| 1.45 – 3.25 | 46 (50.5%) |
| More than 3.25 | 15 (16.5%) |

SD = standard deviation
