# Supplementary material for: Health Disparities in Hepatitis C Screening and Linkage to Care at an Integrated Health System in Southeast Michigan
Source: PLoS One. 2016 Aug 15;11(8):e0161241. doi: 10.1371/journal.pone.0161241 (PMC4985134; doi:10.1371/journal.pone.0161241)
Supplement: S5 Table — (DOCX) [file pone.0161241.s005.docx]

**S5 Table. Full multivariate analysis on variables associated with hepatitis C linkage to care**

| **Variable** | **P-value** | **Odds Ratio** | **Odds Ratio 95% Confidence Limits** | |
| --- | --- | --- | --- | --- |
| **Female Gender** | 0.08 | 2.36 | 0.90 | 6.25 |
| **Electronic Health Engagement** | 0.01* | 3.89 | 1.31 | 11.54 |
| **Income > $40,000** | 0.53 | 1.39 | 0.51 | 3.79 |
| **Medicaid insurance** | < 0.05* | 0.16 | 0.16 | 0.97 |
| **Number of Office Visits** | 0.12 | 1.60 | 0.89 | 2.85 |
| **Charlson Comorbidity Index** | 0.58 | 1.10 | 0.78 | 1.55 |

* Statistically Significant, P < 0.05
